# What’s Next: Recruitment of a Grounded Predictive Body Model for Planning a Robot’s Actions

**DOI:** 10.3389/fpsyg.2012.00383

**Published:** 2012-10-08

**Authors:** Malte Schilling, Holk Cruse

**Affiliations:** ^1^International Computer Science InstituteBerkeley, CA, USA; ^2^Center of Excellence ‘Cognitive Interaction Technology’, University of BielefeldBielefeld, Germany

**Keywords:** prediction, anticipation, recurrent neural network, internal body model, internal simulation, minimal cognitive system, robotic architecture

## Abstract

Even comparatively simple, reactive systems are able to control complex motor tasks, such as hexapod walking on unpredictable substrate. The capability of such a controller can be improved by introducing internal models of the body and of parts of the environment. Such internal models can be applied as inverse models, as forward models or to solve the problem of sensor fusion. Usually, separate models are used for these functions. Furthermore, separate models are used to solve different tasks. Here we concentrate on internal models of the body as the brain considers its own body the most important part of the world. The model proposed is formed by a recurrent neural network with the property of pattern completion. The model shows a hierarchical structure but nonetheless comprises a holistic system. One and the same model can be used as a forward model, as an inverse model, for sensor fusion, and, with a simple expansion, as a model to internally simulate (new) behaviors to be used for prediction. The model embraces the geometrical constraints of a complex body with many redundant degrees of freedom, and allows finding geometrically possible solutions. To control behavior such as walking, climbing, or reaching, this body model is complemented by a number of simple reactive procedures together forming a procedural memory. In this article, we illustrate the functioning of this network. To this end we present examples for solutions of the forward function and the inverse function, and explain how the complete network might be used for predictive purposes. The model is assumed to be “innate,” so learning the parameters of the model is not (yet) considered.

## Introduction

The capability of not only reacting to actual stimuli, but also predicting future stimuli, was for a long time attributed to “higher animals” and therefore tightly connected to properties of (some) vertebrate brains. Now, however, not even “simple” animals like insects are considered merely reactive; it is now known that they are able to anticipate future situations. Anticipation, i.e., the use of information about what will be next, is used to guide actions. Examples include the prediction of the future position of a moving object, which can be used to visually pursue or reach for it, and the estimation of the mass of an object to be lifted. To allow for such prediction, internal models of the environment are required. Seen from the brain’s point of view (Cruse, 1999), an essential part and a starting point is a model of the body. Therefore, internal models not only refer to objects in the external environment, but also have to include a simulation of – at least parts of – the body.

Usually, two types of models are distinguished (Kawato, 1999, p. 718):

“Internal models are neural mechanisms that can mimic the input/output characteristics, or their inverses, of the motor apparatus. Forward internal models can predict sensory consequences from efference copies of issued motor commands. Inverse internal models, on the other hand, can calculate necessary feedforward motor commands from desired trajectory information.” Here, we add a third function, namely sensor fusion and want to explain these function in more detail.

### Inverse models

Classical paradigms for inverse models are targeted and goal-directed movements that fundamentally rely on an internal model. The simple ability to grasp an object seems to be carried out without any explicit planning of the movement but by application of controllers using sensory feedback. But the action is not merely controlled through visual feedback. Targeted movements can be accomplished without sight and so fast that a feedback control loop, which inevitably would include certain delays, would be too slow to account for the behavior (Miall et al., 1993; Desmurget and Grafton, 2000). As a possible solution to this problem, it has been assumed that the controller implements a transformation of the target description onto the actuator dynamics. The target position – given through visual input – may be defined in an egocentric Cartesian space. To reach the target, the position, and the reaching movement must, however, be described in terms of joint or muscle activations in some form. A transformation between these two reference systems represents a mapping from Cartesian space to joint space. This is called an inverse model (Wolpert and Kawato, 1998).

Solving this inverse kinematic problem is difficult because, as is the case in most if not all biological control problems, the controlled system, in our case the limb, contains extra degrees of freedom (DoF), i.e., more joints than necessary for the solution to the task (Bernstein, 1967). This “ill-posedness” means that there is not only one but many solutions. Therefore, the controller has to select one out of these many possible solutions.

Visually guided reaching movements have been the subject of many studies in humans (Castiello, 2005; Shadmehr and Wise, 2005), as well as in other animals. But targeted limb movements can be found also in insects. An example is the optomotor response in crickets. The antennae of crickets can follow moving targets that are visually recognized (Honegger, 1981). Another example is the targeted leg movement in locusts that can be elicited by a tactile stimulus. When stimulating a locust by touching its forewing with a paintbrush, the animal will react with aimed scratching movements, usually of the ipsilateral leg (Matheson and Dürr, 2003; Page et al., 2008). In walking stick insects the swing movement of a leg aims at the current foothold position of the anterior leg (Cruse, 1979). All these aimed movements rely on a connection between sensory information and muscle activation. This mapping solves the inverse kinematic problem and therefore establishes an inverse model.

### Forward models

As mentioned, motor control in general requires feedback information to guide a movement. The whole cycle of motor control, for example the movement to a target, is affected by disturbances, such as misperception of the target position or the target distance and noise in the signal conductance from sensors or toward the actuators. To counteract all these disturbances, sensory feedback is required to supervise the movement, detect deviations from the intended movements, and adjust the control signal. However, in fast movements the controller cannot rely solely on sensory feedback to guide the movement because of delay inherent to the sensory and motor pathways. The question arises: how it is possible that humans as well as other animals actually are capable of such fast movements? A possible solution is that humans predict sensory consequences instead of waiting for their real values. Therefore, control of movements, in particular fast movements, relies crucially on the ability to predict sensory and motor consequences.

A solution for a fast prediction of the real feedback could be provided by a forward model (Miall et al., 1993; Desmurget and Grafton, 2000) as forward models can be used to determine spatial location when joint angles are given. Combined with an inverse model of the body, a forward model can detect a possible error more quickly than one that relies only on proprioceptive feedback. When participating in dynamical tasks, such as catching a ball, an actor must be able to predict the movement of target objects, and therefore must have a forward model of parts of the world that forecasts future states from the current state.

Today, there are many lines of evidence supporting the existence of such models in the brain. Especially for manual or bimanual tasks in humans, much work has been devoted to the influence of prediction on control tasks (Wolpert and Ghahramani, 2000; Wolpert and Flanagan, 2001). An experiment by Strauss and Pichler (1998) suggests that the fruit fly *Drosophila* is able to construct a dynamic representation of a steadily moving optical pattern that disappears behind an occluder. As a consequence, the pattern is expected to appear again on the other side of that occluder. Li and Strausfeld (1999) have found evidence suggesting that the mushroom bodies in crickets differentiate between stimulation as a consequence of intended motor actions and stimulation as an external imposed stimulation. Webb (2004) reviews further examples that involve predictive models and could be termed forward models, such as those that stabilize the visual field in flying insects.

### Sensor fusion

A distinctive feature of animals and humans is the large number of sensors for each modality. This multitude of sensory channels is in sharp contrast to technical systems, which usually use only a handful of different sensors measuring disjunct qualities. In animals, many sensors measure the same or closely related features of the environment, but in different ways.

Each sensory channel may employ its own way of “representing” information. For example, a position of an arm may be described by the visual system in a Cartesian and body-centered coordinate system, while proprioceptive sensors use some kind of muscle length or joint angle-like representation.

A recent review Makin et al. (2008) concluded that a representation of the hand’s position relies on sensory information coming from skin, joints, muscles, eyes, and even ears (Ernst and Banks, 2002). An advantage of redundant systems is that errors due to inconsistencies or to loss of sensors can be canceled out and variances can be compensated for. This presupposes an integration of the sensory information. The integration seems to be realized as a weighted summation of the different information (Makin et al., 2008).

Quite similar results can be found for targeted limb movements in insects. Niven et al. (2010) have shown that desert locusts use vision as well as tactile information from the antennae to guide where they put their limbs when walking on a horizontal ladder. In this situation, the animals are required to make accurate targeted leg placements on rungs to find a foothold, especially when the distance between rungs is variable. On the one hand, the animals directly find footholds for the front legs even when they have not touched the rung with their antennae. The visual information is in this case sufficient. On the other hand, leg placement in insects is strongly influenced by tactile information from the antennae, which is used in searching movements to find footholds for the legs (Dürr and Schütz, 2011). Locusts with occluded eyes are still able to walk over the ladder. Importantly, a deterioration in either modality has a corresponding deterioration in ladder-walking performance.

As mentioned, multiple redundant modalities in a system compensates for errors and disturbances. This, however, presupposes some kind of integration mechanism of the sensory information (see, e.g., Wolpert et al., 1995; van Beers et al., 2002). Such an integration of visual and proprioceptive/tactile information (Botvinick and Cohen, 1998; Muller et al., 2009) requires an internal model of parts of the body, which may be termed a sensor-fusion model and can apparently be found even in animals like insects (Wessnitzer and Webb, 2006).

### Possible neuronal architectures

How might such models be coded neuronally? Recent studies have shown that neuronal systems controlling behavior are constructed in a modular fashion. Flash and Hochner (2005) have reviewed results that lead to the interpretation that “many different movements can be derived from a limited number of stored primitives.” Davidson and Wolpert (2004) demonstrate that internal models underlying grasp can be additively combined. Results of Cothros et al. (2006) suggest that there are distinct neural representations of objects and limb dynamics. Briggman and Kristan (2008) review the arguments for modular architectures, concentrating on the question concerning functional vs. morphological modules. Anderson (2010) reviews a huge body of results supporting the idea of “neural reuse,” i.e., the hypothesis that new modules have been evolved by “massive redeployment” of earlier existing modules.

Specifically, Wolpert and Kawato (1998) proposed a modular architecture, where an individual model is required for each task and each behavioral element. In this approach, not only are predictive and control functions separated, but dedicated modules are used in the context of single behaviors (Wolpert and Kawato, 1998). Such an approach requires a large number of specialized and redundant modules, and excludes the possibility of transferring knowledge between different contexts, e.g., adapting only once to changes of the body geometry or the inclusion of tools into a bodily representation (Maravita and Iriki, 2004).

In contrast, we argue that this type of specialization is not necessary and propose another approach. As each behavior has to be performed with the body, why should separate body models be applied for each of these many procedures? We propose one holistic model that, on the one hand, addresses both control and predictive function, and, on the other hand, which is one core representation that can be recruited by different behaviors and has not to be remodeled in each and every behavior anew. First, we will explain the structure of our model, which is realized as a recurrent neural network (RNN) allowing for pattern completion (Schilling, 2011a). Therefore one and the same model can be applied as an inverse model, for sensor fusion as well as a forward model, i.e., for prediction. An important characteristic of this model is that it can deal with redundant structures, in our case a complex body with 22 DoF arranged in series or in parallel. Complex redundant manipulators are a challenge for many modeling approaches as redundancy allows for multiple solutions and requires some form of decision which solution to choose. For example, the human arm consists at least of seven DoF. Many points close to a person can be reached by many different arm configurations. Instead of introducing an explicit criterion for selecting one solution, in our approach the redundancy is exploited. The complexity of the body is divided into trivial relationships and the Mean of Multiple Computation principle is a mechanism to integrate these multiple relationships. We will not refer to biological structures that possibly reflect this network. Rather, we will use it as a simple example providing a proof of concept for an integrative model that does not need a huge number of dedicated modules. We will also not discuss how the internal body model as such could be learned.

Second, we will explain and discuss how this internal model can be combined with a decentralized architecture consisting of sensorimotor procedures, i.e., be embodied in a biologically inspired control framework for the control of a walking robot (Schilling and Cruse, 2008, submitted). On the one hand, the body model serves reactive control, i.e., the network is applied as an inverse model for the control of the leg movements and as a filter to improve erroneous sensory data. On the other hand, we want to explain how the predictive capabilities of the network can be exploited to anticipate consequences of the application of novel or existing behaviors in – possibly harmful or dangerous – situations. This faculty allows the system to mentally simulate an action before carrying out a possibly unsuitable action in reality. In this way, predictive capabilities of a model can make cognition as planning ahead possible (following the definition of McFarland and Bösser (1993). How the complete model might be used for planning will be discussed in Section “Conclusion and Future Work.” In the Section “Discussion,” we will contrast this approach with approaches in robotics and movement science that rely on a multitude of very specific internal models.

## Material and Methods: The Mean of Multiple Computation Model

In the following, we present a holistic model that can be used in different contexts. This model solves all three problems discussed above. The model is based on an integration principle – the mean of multiple computation (MMC) principle (Cruse and Steinkühler, 1993). The general idea is that the model describes relationships between body parts and that these kinematic descriptions are encoded into a RNN. Although the underlying principle of calculating a mean value between different influences is supported by biological findings on sensory integration (Makin et al., 2008), this network is not meant as a model of one specific part of the brain, nor do we propose that there is one single dedicated body modeling area. Rather, we only want to show the feasibility of such a model as a proof of concept. It is important that the principle proposed for the integration offers to merge multiple sites of information in a coherent way while addressing the three tasks mentioned.

**Figure 1:**
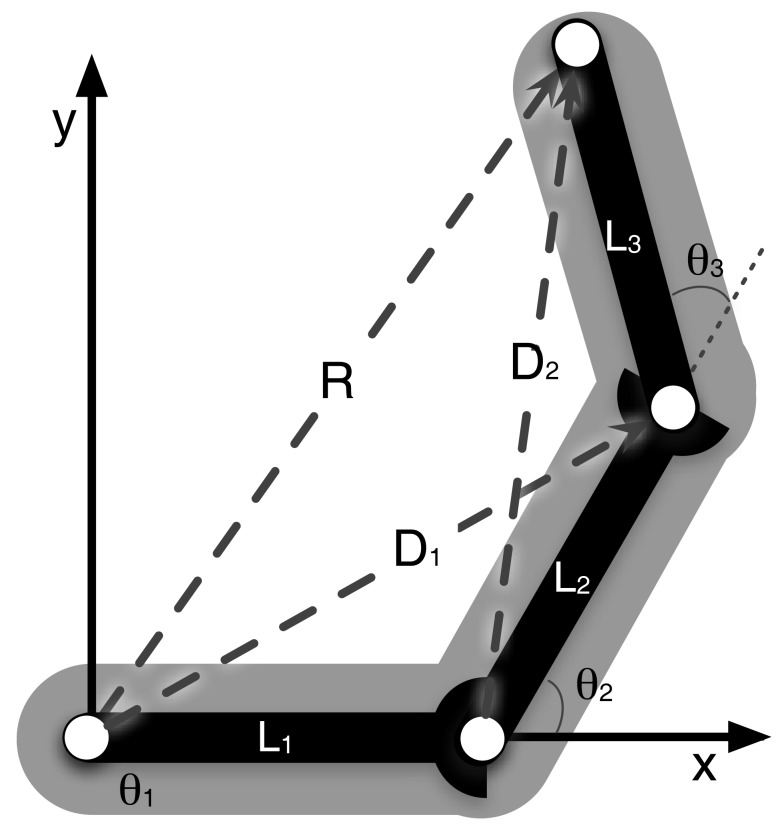
**Arm consisting of three segments (*L*_1_, *L*_2_, and *L*_3_) that are connected by three hinge joints**. The end-effector position is described by the vector *R*. *D*_1_ and *D*_2_ describe the diagonals. The arm can move in a two-dimensional plane, but has three DoF (joints), one more than necessary.

The core of the network describes the structure of the body to be represented – the network can be directly set up from the kinematic equations. Even a simple manipulator structure (like a human arm) can be quite complex, making a direct mathematical solution impossible. This complexity is a problem for control approaches and is usually circumvented by introducing restrictions. In our model, by contrast, the redundancy of the manipulator is not seen as a problem, but is exploited. When setting up the kinematic descriptions we do not encode a complete solution for the whole structure of the body, but we divide the complexity into smaller structures, which can easily be handled mathematically. This leads to more equations than the minimum number required, but they can be solved and solutions can be found easily. Specifically, the structure is split into relationships between three variables each. A variable is either one that describes a moveable joint and the connected segment, or a newly introduced variable capturing relationships between two other variables. The variables describe local relationships (e.g., the upper arm and the lower arm are two variables that construct a local relationship and form a diagonal vector/variable which connects these two, see Figure 1, *D*_2_). Finding a solution for any of these three variables is straightforward and always leads to a solution. Each variable takes part in several such local relationships (see Figure 1) and in the end we can derive a whole set of such local and simple equations (for the example of the arm, the derived equations are presented in the Appendix). Solving each of the equations for each variable, we get multiple ways of describing each variable through its local relationships: there are Multiple Computations for each variable. Following the MMC principle, the multiple solutions for one variable can be integrated by calculating a (weighted) mean. This leads to an iterative way of calculating new values for each variable. At the same time the set of equations can be understood as constituting a neural network. The introduction of recurrent connections dampens and stabilizes the system as it introduces low-pass properties (the equations describing the resulting network are given in the Appendix, for more details see Schilling, 2011a).

While the multiple computations appear to introduce additional but unnecessary computations, this is true only while the network is in a harmonic state, meaning all the multiple computations for one variable lead to the same result. But when, due to a disturbance, the different computations lead to different values the network basically performs a form of pattern completion. It acts as an attractor network forming an autoassociator and integrates the different solutions in a coherent way constrained by the encoded relationships. This means that the network overall settles into a state consistent with the encoded relationships that basically span the activation space of the network. In this way the network can fill in missing information or correct wrong information. By that means, it can produce solutions for the inverse, forward, or any mixed task.

### The MMC body model

In the following we want to explain how such a network can be setup as a body model for a simple animal such as a six-legged stick insect. We will start with the description of the kinematics of a single leg, which is comparable to the example of an arm. In the next step we will extend this network toward a model of the whole body, showing how different levels of representations can be integrated and how the model mediates between the different partial models. To this end, we show how this complete model can be applied in motor control and how a leg model can be utilized for the inverse model function in this task. Later, we will discuss how this model can be used for planning ahead.

**Figure 2:**
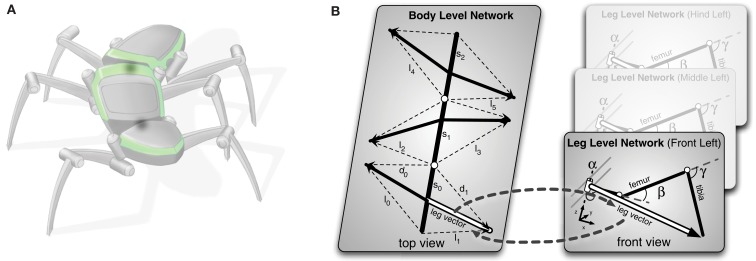
**The hierarchical body model**. In **(A)** the vectors constituting the higher (body) level of the model are shown. Each leg is only represented by vectors to the end point of the leg. The detailed geometry is not reflected on this level. In **(B)** it is shown how the two levels are connected. Each leg is represented through a single leg network as shown in Figure 3. Each leg network shares the end point vectors with the higher body level network. During processing both levels mutually inform each other. In the inverse kinematic case the body level produces new leg vectors as target vectors that are forced onto the leg network, which comes up with corresponding joint angles for the target vectors.

The complete model has a two-layered structure (see Figure 2). The lower level contains six models, one for each leg (Figure 2B, right). The upper layer represents the thorax and the six legs, the latter, however, in an abstracted form (Figure 2B, left). We will begin with describing the model of the individual leg.

#### The leg model

**Figure 3:**
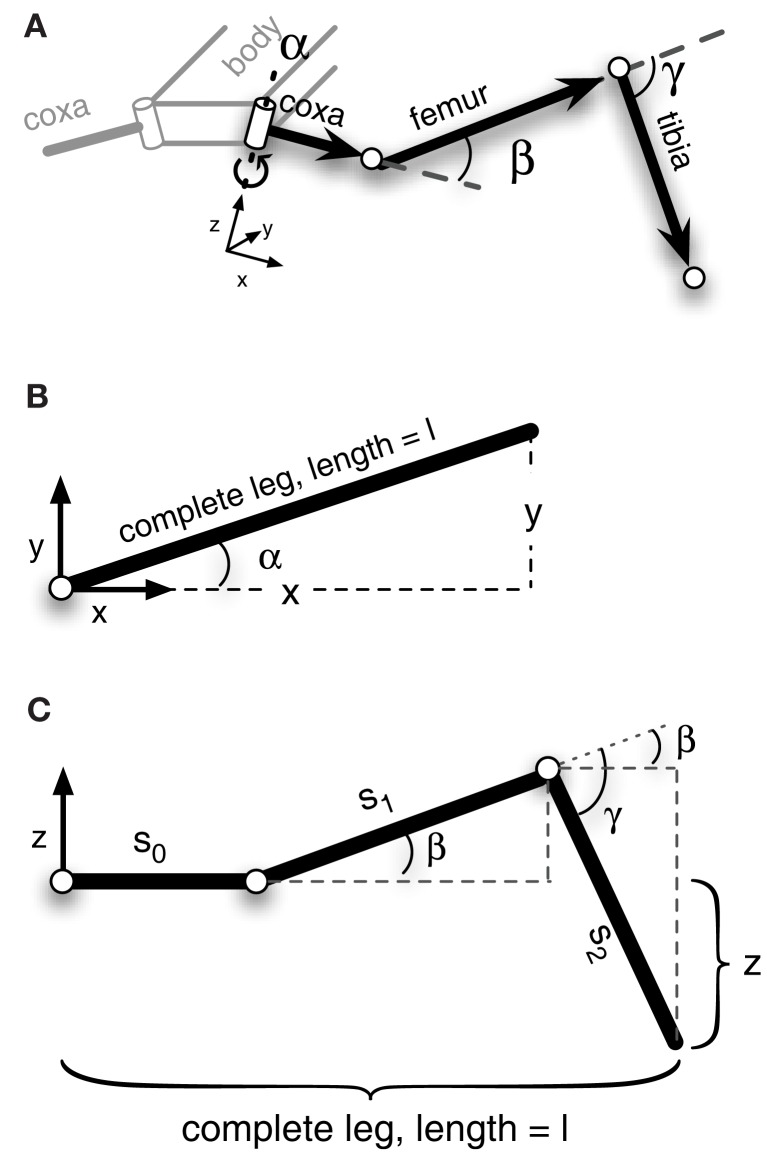
**Schematic figure of the left front leg of a stick insect**. The leg consists of three hinge joints. **(A)** Shows a view of the complete leg attached to the body. **(B)** Top view of the leg. The α-joint moves the leg forward and backward. In **(C)** the leg is seen from the front. β- and γ-joint operate on a plane, meaning their axes are parallel to each other and are perpendicular to the leg plane. Lifting the leg equals a positive movement in the β-joint, and an outward going movement produced by the γ-joint is defined as a positive movement. The origins of the joint coordinate systems are set corresponding to leg positions in a standing walker (α is in a middle position, while β- and γ-joints are in a position in which the femur is approximately parallel to the ground and the tibia is nearly orthogonal to the femur).

Figure 3 shows the structure of a stick insect’s leg that has been modeled. It only contains three DoF. We can set up a simple MMC network using redundant trigonometric relationships. Because of the kinematic structure, we can derive a specific solution for this type of manipulator. As the second and third joint act on a plane (Figure 3C) and their rotation axes are parallel, we can use basic trigonometric function to come up with a solution for these joint angles that hold true in this plane. The first joint angle can be derived from the projections of all leg segments on to the ground plane. Even though for this kind of structure a closed mathematical solution is possible, we restrict our solution to simple trigonometric relationships. This leads to multiple computations of the variables that can then be integrated into the model (more details on the derived equations are given in the Appendix).

As the model directly encodes the kinematic equations describing the structure of the leg, the local relationships basically represent the forward kinematics and in this way provide a means to translate movements of joints into displacements in three-dimensional space. The partial solutions are then combined through the shared connecting variables. When a set of joint values is given, the model adapts its internal values in a complementary way. The result is a leg configuration that is geometrically valid as the network activations are restricted by the encoded geometric constraints (e.g., fixed segment length, joint angle limits). This property is independent of the input given to the net being underdetermined or overdetermined.

While for a single leg the number of DoF is quite limited, the model as such is not limited in this respect and the MMC principle can and has been applied to model manipulators with many more DoF. It had been applied to three-segmented manipulators in general and it has been shown that it can be used in such scenarios with universal joints and nine DoF in total (Schilling, 2011a). The model can as well cope with additional constraints applied to it, for example, when modeling a human arm with seven DoF for the whole arm and an elbow joint that is restricted to movements in one dimension (Schilling et al., 2012).

#### The thorax model

When we want to look at the more complex case of a whole body, which for the insect corresponds to three body segments and six legs, we can divide the complexity of the problem into meaningful levels (Figure 2). To this end, the model is constituted of detailed models of the individual legs, as described above, while for the complete model of the whole body in the upper layer, the thorax model (Figure 2B, left), the legs are only abstracted to the vectors representing the end points of each individual leg (for more details on the representation on the body level see Schilling et al., in press). Such an approach has two immediate advantages. First, it divides the complexity into different levels and therefore reduces on each level the number of involved variables and as a consequence the number of redundant derived equations to a manageable set. Second, it introduces a form of explicit abstraction that is reflected in the structure of the model.

**Figure 4:**
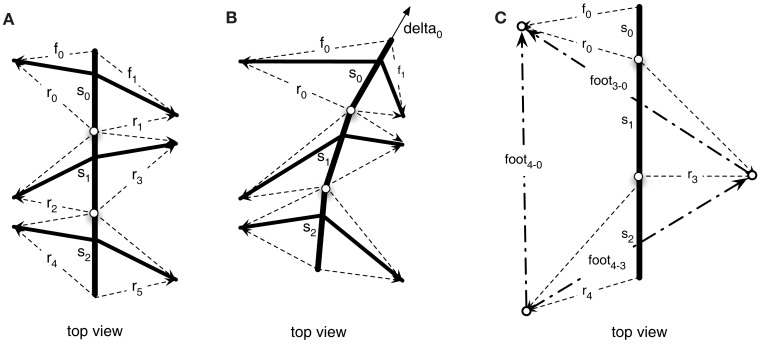
**Vectors constituting the thorax model (view from above)**. In **(A)** the vectors describing the foot point with respect to the segments are shown. **(B)** Shows how these vectors are changed when the model is pulled at the front (delta vector) and the foot points are kept in place. **(C)** Shows an example configuration during walking, with only three legs on the ground (front left, middle right, hind left).

The different levels of the body model are connected as they share variables, in the case of the insect the vectors pointing to the tip of the leg. The computation of the different levels is tightly interwoven through these shared variables. This allows the model to be flexibly used in different scenarios. For example, we can use the body model to control the coordinated movement of the legs during the stance movement in forward and curved walking (Schilling et al., in press). In the upper level (Figure 4A), we initiate the movement of the body by pulling at the front segment (see Figure 4B, vector delta_0_), while the other segments as well as the legs pick up the movement. Through the shared variables, the movement of the leg in the thorax model is given as an input to the leg networks and the leg networks provide the complementing joint movements for motor control.

### Procedural memory elements and motivation units

The body model as such is not able to create specific behaviors. Its main function is to filter input data in such a way that the resulting output corresponds to the geometrical (and, in the extended version Schilling, 2009, dynamic) side conditions given by the body. To drive specific behaviors, a bank of procedural memories is required (see Figure 10 for an overview of the decentralized control system for a single leg). Examples are given by a network called Walknet (Dürr et al., 2004) which, being based on behavioral studies on stick insects, produces descriptions of many complex behaviors (such as climbing over a gap that is wider than twice the step length of the animal Bläsing, 2006). The most important procedures with respect to walking concern the Swing-net and the Stance-net, controlling swing movement and stance movement, respectively. Both procedures exploit sensory feedback, joint angle position, or velocity to provide angular changes to be performed in the next moment of time. In the case of the Stance-net, the contribution of the individual joints is determined by the body model.

To control the temporal sequence underlying any behavior, for example the more or less regular sequence of swing and stance movements involved in walking, an additional neuronal structure is required. Inspired by Maes (1991), who was herself inspired by Konrad Lorenz, we equip each procedural element with a motivation unit that gates the output of the corresponding procedural element. These motivation units form a separate network as they may be coupled with mutual excitatory or inhibitory connections. This network can adopt a number of stable (attractor) states that provide the context for a specific procedure to be selected. In the examples, to allow for a simple explanation of the principle, we use only the Swing-net and a second procedure, Reach-net, explained below, together with their motivation units.

## Results: Simulation of the MMC Network

We will show two sets of simulations. The first one (application as a forward model) demonstrates the predictive capabilities of the MMC network. The second simulation demonstrates how the same network can be used in motor control to make targeted movements (application as an inverse model).

### Application as a forward model

**Figure 5:**
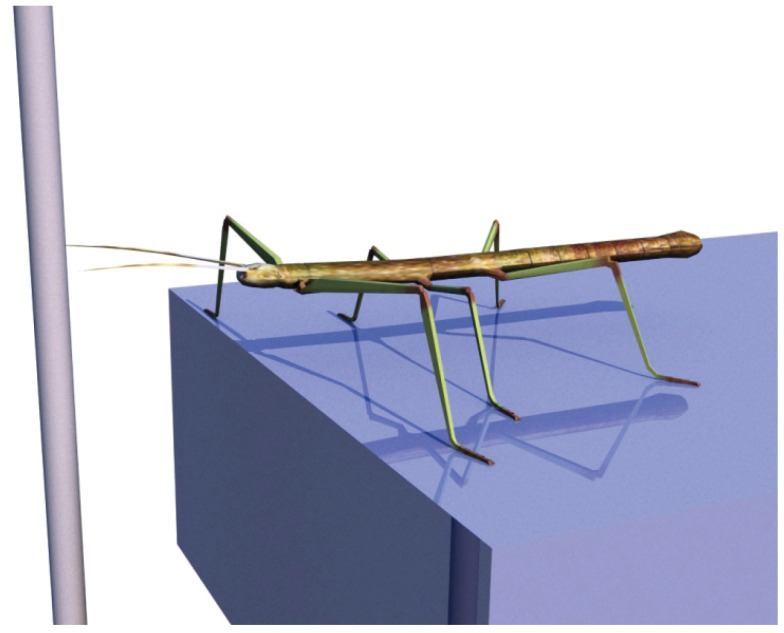
**Insect in front of a gap**. Left front leg will perform a searching movement that is controlled by the Swing-net. The leg network can be used in this case to estimate the end position of the leg (forward function).

**Figure 6:**
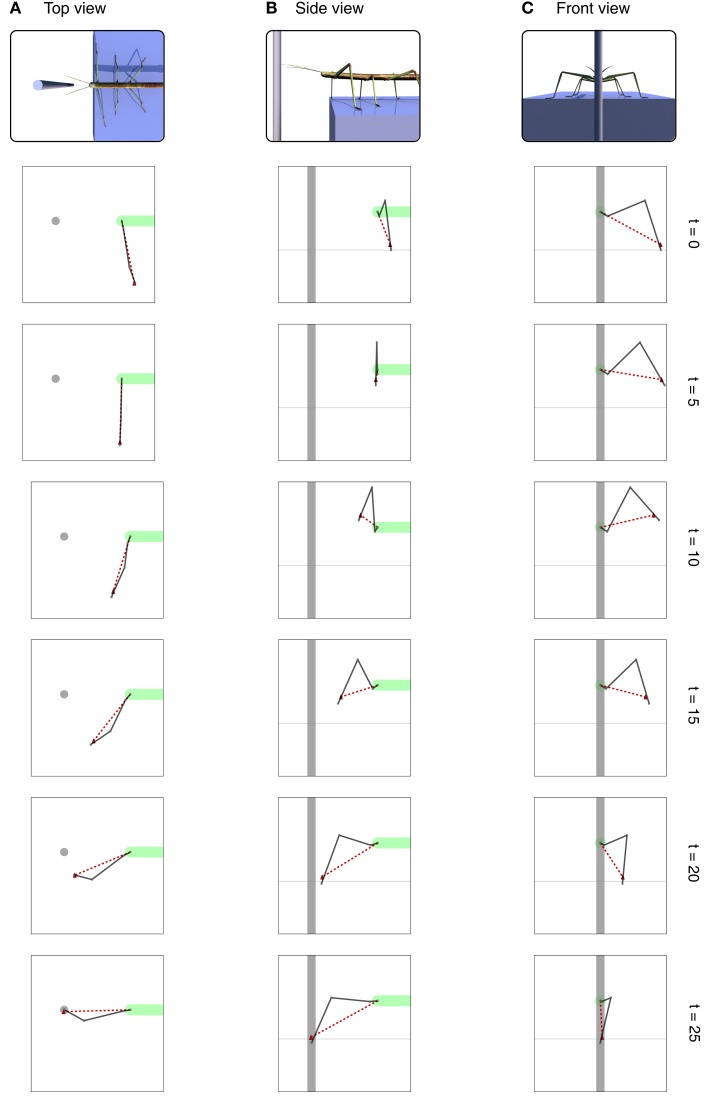
**Different views of the movement of the leg during the search movement**. The dark solid line always shows the current leg configuration as described through the joint angles. The red dashed line shows the position of the tarsus as estimated by the MMC leg network. The horizontal dashed lines in **(B,C)** indicate the ground level. View from above is shown in **(A)**, side view is shown in **(B)** and view from the front is shown in **(C)**. Right: number of iterations.

To illustrate the basic function of the model, we will consider the scenario of a walker climbing in an environment on footholds that are sparsely distributed. Specifically, we assume that the walker is standing in front of a gap where a vertically oriented beam is positioned in the sagittal plane of the body and near enough so that the beam could be reached by a front leg (see Figure 5). We assume that the animal (or robot) does not exploit visual input nor does it use tactile input from the antennae. When the walker continues walking, it uses a procedural memory element called Swing-net. This network provides signals for how to move the joints during a swing movement. The latter is characterized by a trajectory that describes a movement forward that involves a lifting movement in the first part followed by a downward movement in the second part of the trajectory. In normal walking over flat terrain the swing movement ends as soon as the leg touches ground. In some versions of the Swing-net (Dürr, 2001; Bläsing, 2006), a somewhat regular searching movement is performed if no ground contact is given. During a swing of an insect standing in front of a gap, where only the vertical beam can provide ground contact, the leg may be moved until it finds a possible support at the vertical beam. Note that the body model does not contribute to control this swing movement. Nonetheless, during the swing movement, the actual values of the joint angles are given to the RNN forming the leg model, thus disturbing its actual state. As described above, this leg network starts to distribute the externally introduced disturbance onto all variables that are part of the network. As a consequence, all variables adopt values that complement the ones forced onto the leg network. As the network acts as an autoassociator, and as all the values are restricted by the encoded geometrical and kinematic structure of the modeled body, the network also contains the vector describing the end position of the leg. This information will be exploited in the second example explained below. Figure 6 shows a simulation run in which the front left leg is making a swing movement driven by Swing-net. Shown is the real configuration of the leg as given through the joint angles (solid lines in the figure) as well as the vector pointing to the tip of the leg (dashed lines). As the figures show, the leg position estimated by the body model is quite close to the real position. Thereby the network solves the direct kinematic task.

**Figure 7:**
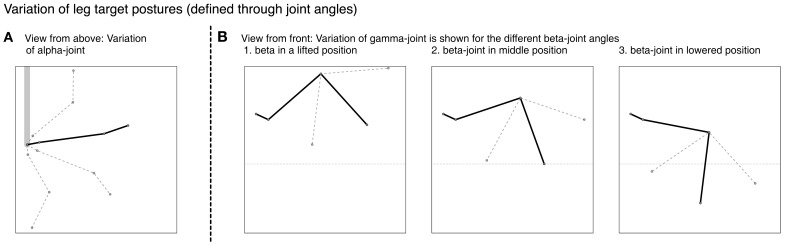
**Different leg postures – produced through variation of joint angles**. In **(A)** the four different alpha joint angles used for the definition of the postures are shown. In **(B)** the three different figures show the different postures stemming from the variation of the beta joint, each showing the three gamma joint values applied.

In the example given in Figure 6, we showed how the body model is able to determine the end position of the leg during a swing movement. To give an impression concerning the behavior of our model, we test how well the vector pointing to the tip of the leg corresponds to the actual position determined by the joint angles. Therefore, we tested our model on a number of movements between 36 pre-defined postures (see Figure 7). These result from four different joint angles used for the alpha joint (87, 37, −13, −63°), three variations for the beta (15, 40, and 65°), and the gamma joint (36, 86, and 136°).

**Figure 8:**
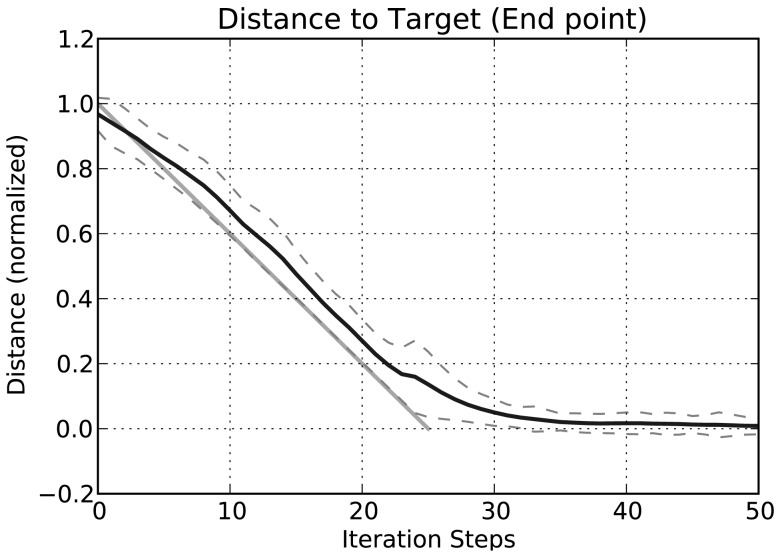
**Distance of the estimated leg end position to the position of the target posture in three-dimensional space over time**. The mean normalized distance is calculated for all 1260 movements for each iteration step. The distances are normalized with respect to the distance between start and end posture in three-dimensional space (dashed lines show the SD around the mean value). The light gray line indicates a linear interpolation between the start and the target position (Importantly, the interpolation is done in joint space with a constant velocity. As a result, the interpolation of a single movement is better described by a curve (a geodesic), but over all movements we use a straight line as a simplification to indicate the general expected movement characteristics).

In 1260 simulation runs in all, we now produced movements from each posture to every other posture. Initially, in each run the network is provided with the joint angles of the start posture as an input and iterated for 100 iteration steps, so that the network is in a settled state and represents the start posture adequately. Then the actual test begins. For 25 time steps, each joint is moved from its start angle to the target angle. The joint angles change linearly over time and these joint values are used as input to the leg level of the body model, which is iterated as input is provided. The body model predicts the end position of the leg. Figure 8 shows the Euclidean distance between the predicted end point and the target point over time. This distance is normalized with respect to the overall distance between the starting point and the target point. As can be seen from the figure, the body model follows nicely the imposed movement. There is an expected time lag as the used model does not anticipate the continuation of the movement, but merely integrates the current sensory data into the old estimated position and therefore underestimates the overall movement. (In an extension of the MMC network, we introduced dynamic influences and integrated equations representing velocities and accelerations in the network. As an effect, such a network can also successfully predict the ongoing movement and the lag is reduced correspondingly; Schilling, 2009. Including dynamic influences also counteracts the exponential slowing down at the end of the movement.) After 25 additional iteration steps the body model has settled close to the target position. The mean distance between target position as given through the joint angles and the estimated end position of the leg provided by the body model is 0.1598 (SD ± 0.112) at iteration step 25 (when the movement of the input is finished) and 0.0084 (SD ± 0.026) at iteration step 50. This is a normalized distance with respect to the overall distance between start and target position. A side effect of this normalization is that some movements that actually are quite close in three-dimensional space nonetheless require substantial movements in the joints. In such cases the normalized distance over time gets inflated by the normalization process. Looking at individual results we found that small positional differences between starting and target posture had substantially higher normalized distances, which increased the error measurement and the SD.

### Application as an inverse model

In the next simulation, our goal is to demonstrate how the internal body model can be used as an inverse model. We show that after the left front leg has found foot contact on the beam (see Application as a Forward Model), the contralateral, right front leg can make a targeted movement to the same spot at which the left front leg found a foothold. The left leg was driven by a simple behavioral module, Swing-net (see first simulation in Application as a Forward Model), and we used the body model to estimate its position. In the next step, the contralateral, right, leg should aim for this position. The information transfer between these two legs is mediated via the upper level of the complete body model (see The Thorax Model). Parts of the body model are vectors describing the relative position of the tips of all legs (see Figure 4C). For example, in Figure 4C, vector foot_3–0_ connects the foot of the left front leg (#0) with that of the right middle leg (#3). Correspondingly, vector foot_1–0_ (not shown in Figure 4C) connects the left front leg with the right front leg (#1). Therefore, to control a direct, targeted movement of the right front leg toward the current position of the left front leg, we need another procedure, termed Reach-net, that simply sets vector foot_1–0_ to zero and thereby enforces the body network to adopt a foot_1–0_ vector of length zero. In this way, the body network will generate a new target vector for the right front leg which is then given to the lower-level leg network. As the network has to satisfy this constraint, the right front leg of the model will approach the position of the left front leg, thus solving the inverse kinematic task.

**Figure 9:**
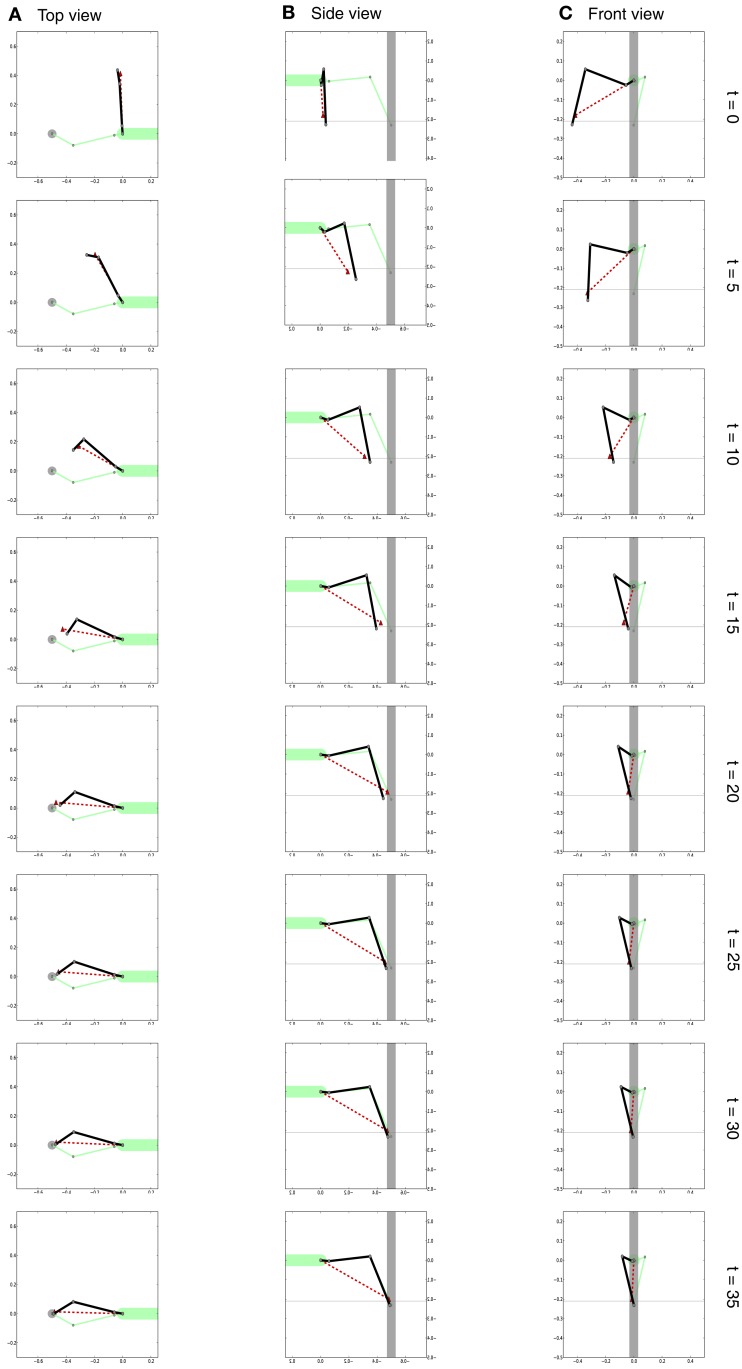
**Different views of the movement of the right front leg (dark solid lines) during the targeted movement toward the front**. [Views as shown in Figure 6. Note that as we are looking at the right leg in **(B)** we are looking from the other (the right) side.] Shown is the movement over time. The configuration of the left leg is shown as a solid light green line. The red dashed line shows the target position provided by the body model as a target vector for the leg network of the right front leg. View from above is shown in **(A)**, side view is shown in **(B)** and view from the front is shown in **(C)**. Right: number of iterations.

Figure 9 shows a simulation run. The position of the left leg touching the beam is given by solid gray lines. At *t* > 0 Reach-net is activated, which changes the target position for the right leg to the current position of the left leg. This change in target position is mediated by the upper level of the body model and depicted by the dashed line. As a consequence, the right leg (dark solid lines) is reaching for the target position and is therefore moved into the direction of the target position. The leg is moved to the front through a movement of the first (the alpha) joint and then reaches out to the target position by moving both the second and third joint. In a couple of iteration steps the leg closes in on the target position and touches the beam meeting the left leg. The leg network is able to provide matching joint angles for a given target position and in this way solves the inverse kinematic problem.

## Discussion

The MMC network can be recruited as a body model in diverse tasks as it serves different function. The body model can address the three functions of forward modeling, inverse modeling, and sensor fusion. We have used similar models in the past to solve the inverse kinematic (Schilling, 2011a; Schilling et al., 2012) and inverse dynamic (Schilling, 2009) problems. In this article we showed how the model can serve as a forward model and predict from motor commands given as joint angles (or movements) goal positions of legs in Euclidean space. In the following, we first discuss how our approach compares to other approaches employing internal models and approaches to solving the inverse and forward kinematic problem. Second, we address how the model will be embedded in our control framework reaCog (Schilling and Cruse, submitted). There, due to its flexibility, the model can serve all functions of an internal model. In particular, the predictive capabilities allow recruitment of the model in planning ahead and use of the model as a grounded internal representation to anticipate action consequences. We will discuss connections to other motor control approaches utilizing internal models for prediction in the sense of planning ahead.

### Internal body models

An important notion in the context of motor control is the internal body model, a representation of an organism’s own body and its environment. Even though the work on embodiment has pointed out that complex behavior is possible without an explicit representation and can rely on the “body itself as its own best model” (Brooks, 1991), the intention was not to abandon internal representations, but to focus on grounded internal representation (Steels, 2003). Following this line of research, internal models have to be in service for some lower-level function or behavior before they can be used in a different context. One important part of such a model is a model of the body (Cruse, 1999) as it provides a starting point for models of the environment, i.e., the way the environment relates to an organism’s body. The MMC model is an example of such a model that, at first, can serve behavior (targeted movements), but then is flexible enough to allow for prediction and sensor fusion and in this way may be employed for planning ahead (see Internal Models Used for Planning Ahead Through Internal Trial-and-Error).

Until now, we have focused in this paper on the forward function of the model and how this allows predictions of consequences of actions. In the following, we briefly discuss the properties of the proposed model with respect to aspects of internal models as raised by Haggard and Wolpert (2005). According to them, the term “body schema” stands for the unaware spatial coding of body parts (Paillard, 1999) and is comparable to our notion of an internal body model. (In contrast, the term “body image” is a visual and conscious representation of the body seen from the outside.) In the following, the different aspects (we leave out those related to phenomenological experience) are listed together with an explanation of how they refer to our MMC model:

- *Spatially coded*: The internal model represents the body and the configuration of the body. In our MMC network, the configuration of the whole body can be described by the joint configuration. The positions and relations in space result from the forward kinematic function.
- *Modular*: The brain is assumed to represent the body in a modular manner and in different neural modules (Imamizu and Kawato, 2008). The different modules must be able to interact. Hierarchical MMC networks allow a representation to be modularized easily. The complexity can be distributed on different levels of the hierarchy. The different layers of the network can cooperate by using shared variables describing their geometric relations.
- *Updated with movement*: Haggard and Wolpert (2005) demand that a body model used for the production of action has to continuously track positions and states of the body segments. It is essential for our approach to use the body model as a central part of the whole architecture. The MMC principle is basically an integration principle that allows a value for a variable to be derived from multiple values and influences. In the same way the system can be extended and used to integrate more influences and directly integrate sensory data. For a detailed discussion about how the body model can be used for sensor fusion see Schilling (2011a) or Schilling and Cruse (2008).
- *Adaptable*: Until now, the presented body model does not account for changes of the body geometry or learn even the loss of a leg. The body model is assumed to be innate and may later be modified by experiences and adapted to bodily changes (Funk et al., 2005).
- *Supramodal and interpersonal*: There are distinct areas in the brain that are responsible for processing sensory data from a single modality. The information from the different modality-specific regions is integrated by association areas (Gallese and Lakoff, 2005). The body schema is referred to as such an association area, where the integration of sensor data from different sources is an essential aspect. The MMC principle provides a basic mechanism through which multiple inputs and influences can be integrated and which could be applied there.
- Haggard and Wolpert (2005) further propose that the body scheme is not only used to represent one’s own body, but also to represent the bodies of others. In a scenario with two agents we applied the body model for perception and control of action (Schilling, 2011b). One agent was making targeted arm movements using the body model to provide motor commands. The second agent observed the movements from a fixed point of view. The movements resulted in postures lying in the viewing plane of the observing agent. Lower-order visual moments were used to represent the visual input. It was the task of the observing agent to predict these visual descriptors from the current stream of sensory data. We used a RNN for this prediction consisting of one hidden layer. The structure of the hidden layer was fixed and the hidden layer was identical to the body model used for production of the movements. The observing agent was able to learn the input and output mappings in an unsupervised fashion. The dynamics of the hidden layer were exploited to reproduce the dynamics of the observed movement and to predict the movement correctly. This is a first step toward a multimodal representation. A mapping of the visual impression of another body onto one’s own body model is established (Schilling, 2011b). As the body model is utilized in action and perception it provides a connection between action representation and perceptual effects as proposed by the common coding theory (Prinz, 1997).

In contrast to our approach, various authors have tried to address kinematic problems through individual models. In an early and interesting approach, Morasso and Sanguineti (1994) connected the individual models for the inverse and forward kinematic function. The output of the inverse model was routed to the forward model and vice versa. In this way, a RNN is constituted which is able to perform pattern completion similar to our approach. But it presupposes forward and inverse kinematic models, which may be hard to learn for complex structures. The advantage of the MMC approach is that it is based only on simple local relationships.

Other approaches to implementing forward and inverse functions usually separate both functions and employ independent models for each function. A classic example of such models is the MOSAIC model, which proposes pairs of inverse and forward models to represent individual motor programs. A single motor primitive (a procedural motor program representing the controller of a behavior; overall the motor primitives constitute the motor memory) is defined through the inverse model, which captures the dynamic relation between a goal state and the corresponding motor commands (Thoroughman and Shadmehr, 2000). In the case of targeted movements a goal position is described in Euclidean space and the inverse model would provide movements of the individual joints as motor commands. A motor primitive following the MOSAIC approach consists of a collection of such inverse models, each one paired with a forward model. While both models can be learned at the same time, the main function of the forward model is to offer a prediction of the currently issued motor commands. This prediction can be, first, used as a prediction of the slower sensory feedback. Second, the prediction can be later compared to the actual feedback the system receives. When the predicted value and the actual feedback are in good agreement, the respective model is modeling the current behavior well. Because in the MOSAIC framework these pairs of models are used in parallel and predictions are derived for all forward models, the comparison can be used to choose the current behavior. Therefore, the advantage of such pairs of forward and inverse models as well as learning them in combination is that the switching of motor primitives can be directly linked to the motor primitives themselves. Each motor primitive provides a measurement of how good that behavior fits the current context.

This is in contrast to the architecture we use, in which all motor primitives compete in a winner-take-all fashion (on the level of motivation units) about which primitives should be active, although merging of procedures is not precluded. The activation of a motor primitive is given through the situational context that depends on current sensory states and the current internal state of the system. One important problem for control frameworks in general is adding new behaviors. In the case of the MOSAIC controller, it is hard to decide when a behavior should be regarded as a new behavior or when it should just be understood as a variation of an existing behavior (e.g., reaching in a different direction). While this problem holds true in the same way for our approach, in the abstraction we introduced through the higher level of motivation units, quite complex and adaptive motor primitives may be built on top of the lower level, which can simply be separated by sensory signals.

In the DAC series of robots, Verschure et al. (2003) introduced a hierarchy of abstraction levels similar to ours. In his approach, the lower-level motor primitives were learned together with a more high-level and abstract representation that basically defines in which context a behavior should become active. Learning a motor primitive would be possible in the same way for our system, but currently our system consists of a pre-defined set of motor primitives that are biologically inspired from experiments on the walking of stick insects.

A serious disadvantage of the MOSAIC control framework compared to our approach is the enormous redundancy of the information. For each behavior a new pair of forward and inverse models has to be learned. Each of these models has to incorporate all the aspects required by Haggard and Wolpert (2005) as listed above, i.e., each model has to capture the basic geometric constraints and relationships and basic assumptions concerning the dynamics of movement. Not only would such a redundant system be unnecessary as it represents all these relationships multiple (and presumably a large number of) times, but it also would be difficult to adapt changes of body geometry as these would have to be changed in all the dependent models. In our model, changes in body geometry have to be applied only once to the system and not to each and every individual motor primitive. In addition, as argued above, it has been found that internal models are also recruited in perception (Loula et al., 2005) and therefore must be quite flexible and may not be restricted to specific body sizes.

The essential aspect of the MMC model is not constituted by the body dimensions as such, but is formed by the generic geometric relationships between body parts that hold true for other people’s bodies as well. In this sense, the MMC model may only provide a core representation of the kinematic constraints that can be used by different motor primitives.

Such a core representation of the body is supported by experimental findings. A distinction between an internal model of the body’s kinematics or dynamics and task- or behavior-specific models has been found by Cothros et al. (2006). In their experiments, subjects learned targeted goal-directed reaching movements while at the same time holding a robotic-device that applied novel force fields to the arm during the movement. The representation of the dynamics of the behavior appear to be separated from the representation of the body dynamics and kinematics. After adaptation to the force field subjects performed the same movements either in free space or in a null field holding the robot. Aftereffects during movements in free space were significantly smaller compared to those in a null field. Furthermore, no reduction in retention was observed when subjects returned to the force field after moving in free space. The representation of the object-related dynamics appear to be separated from the representation of the body dynamics and kinematics.

Another approach related to ours is the work of Bongard et al. (2006). These authors have used an internal model of the body in a starfish-like four-legged robot. In their system, the internal model was used in internal simulation loops to evolve locomotion controllers. The internal model was used to predict sensory consequences of the generated motor primitives and to access the quality of the resulting behaviors. After learning a suitable new locomotion motor primitive this controller was then applied to the robot itself. From the difference between the predicted outcome of the motor primitive and the result when carried out on the real robot, the system was able to bootstrap over time changes of its own structure and to adapt its internal model of the body. It was, for example, able to recognize the shortening of a leg and to change its internal body model, as well as to adapt the locomotion motor primitive. Such an updating routine of the internal model could be similarly introduced into the way we are applying our model as our model is also predictive. In Bongard’s approach the internal model is predictive and the forward function of the internal model is exploited in internal simulation. In addition, the model is refined over time, but lacks the flexibility of the MMC model as it is only a predictive model that cannot be used for other tasks. In addition, it is not biologically inspired or related to cognitive function as such, but only computes the forward function. Furthermore, the robot structure used consists only of eight DoF and it is difficult to imagine how this approach could easily be applied to a system able to control complex behaviors, as is the case for the insect-inspired hexapod robot.

A different approach has been proposed by Butz et al. (2007); Herbort et al. (2010) based on the SURE_REACH model. SURE_REACH is a posture-based theory (Rosenbaum et al., 1993, 2001) in which a set of postures is stored in neural population codes. Crucial for motor control are two mappings. First, for a given goal state (a hand position) an appropriate posture or combination of postures has to be selected. This requires an inverse model of the goal space to the posture space. The activation stemming from the goal state drives the activity in the posture space. Second, the changes in activity of the posture space can be projected to motor commands. The motor commands invoke the movement and therefore a change in posture which is fed back into the system into the posture space. The SURE_REACH model has been tested for an arm with three DoF acting in a two-dimensional plane. This manipulator is redundant and one of the strengths of this approach is that it can deal with the redundancy. The SURE_REACH model is able to learn the bidirectional mapping between joint and Euclidean space in an unsupervised fashion. It provides a population coding of the sensorimotor mappings that is in good agreement with neuroscientific findings (Doya et al., 2007) and allows for goal-directed movements while avoiding obstacles. Unfortunately, the redundant coding of the complete arm comes with high computational costs as the number of DoF increases. Therefore, in a recent paper Butz and colleagues conclude that model does not scale up to the complexity of nine DoF like in a human arm (Ehrenfeld and Butz, submitted). In consequence, they developed the Modular Modality Frame (MMF) approach in which the overall complexity of the manipulator is distributed onto local relationships between neighboring segments. This is quite similar to the MMC approach as it is based on redundant local relationships. This model is used for the representation and integration of sensory data of an arm. A central idea is that there are redundant representations and that position and orientation of a limb are represented at the same time with respect to multiple frames of reference. Similar to the MMC approach, the model is modular and relies on local relationships between adjacent limbs of the arm. Relative forward and inverse kinematic transformations are computed between adjacent limbs in the model. In addition, representations with respect to a global frame of reference are continuously updated. Each frame of reference can be connected to multiple sensory inputs. The sensory inputs are integrated and as a consequence the network is able to compensate for noise. In addition, the computation of a plausibility value allows the network to account for (systematical) errors of sensors. The MMF model has been introduced to account for sensor fusion and it has been shown how the model can integrate different sensory channels as well as how it can deal with systematic failure. At the same time the MMF model is based on local computation of forward and inverse kinematic computation in a similar way as the MMC network. In the future, we want to extend our MMC model toward multiple sensory inputs and might use similar ideas to realize the sensor fusion in our model (weighting of inputs, plausibility measurement). While Kalman filters (Wolpert et al., 1995) have been widely used for sensor fusion and integrating these values into a current state, a crucial problem of the Kalman filter approach is that it relies on a minimization procedure required by the inverse modeling step for complex manipulator structures. As a consequence, not all states possible for redundant systems can be realized by the system. Only specific solutions are found (Grush, 2004). Again, approaches based on local relationships circumvent this problem.

While both the MMC model and the MMF model are based on kinematic descriptions that are used to set up the model, there are some approaches in which body models are learned as mappings from visually observed movements to motor commands. Most of these models deal with quite simple robotic structures and are applied to robot arms with a small number of DoF (for a thorough review see Hoffmann et al., 2010). One nice example is the work by Sturm et al. (2009), in which a Bayesian network is used. The network identifies the kinematics of the robot arm just through self-observation over time. The model successfully learns kinematic relationships between neighboring segments of the arm depending on the relating joint variables. Therefore, the model is – similar to the MMC model – based on local relationships that can then be combined to construct the kinematics of the whole robot arm. The local models are learned through a non-parametric regression. It is searched for a best arrangement of these models in order to represent the full system. The forward model has then been applied to predict movement consequences and derivations between prediction and observation have been used to adapt the model. In this way the model was able to adapt to changes of the robot dimensions. This shows the feasibility of learning such mappings and has been used even for a manipulator with six DoF. Nonetheless, it appears difficult to scale such an approach to more complex structures like robots that not only consist of a series of limb, but also have parallel limbs, such as a hexapod walker, as the basic considerations provided by Sturm et al. (2009) on the complexity of learning point out. For such a case at least some basic information on the structure of the robot seems necessary.

### Internal models used for planning ahead through internal trial-and-error

We have shown how a specific type of body model, forming a holistic system, can be used as a forward model and as an inverse model. Because it represents a pattern completion system that is restricted to geometrically consistent output vectors (i.e., body configurations), the MMC model can likewise be used for sensor fusion. Forming a redundant representation, the model is able to distribute large errors over the whole system, thus decreasing the effect of the errors. This faculty will not be discussed here further, however.

**Figure 10:**
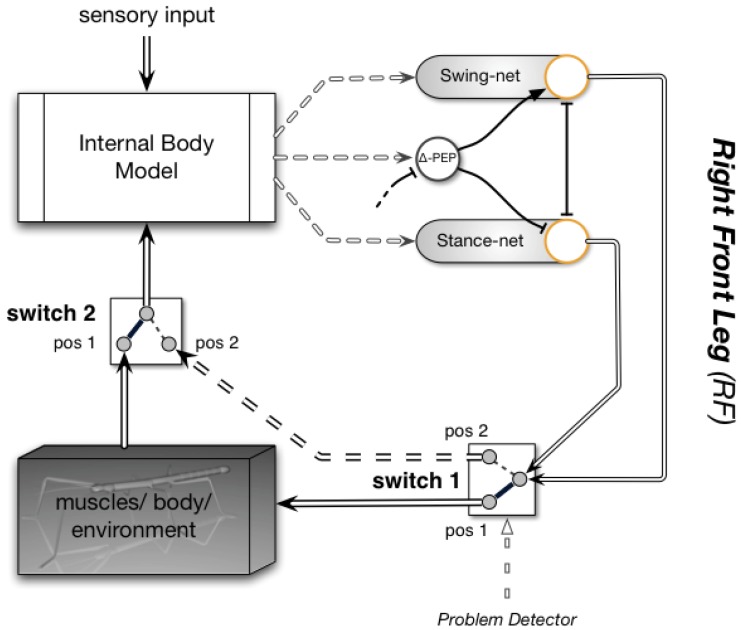
**The first step from the reactive architecture for the six-legged walker to a cognitive architecture: the controller includes an internal body model which is used for sensor fusion (and can be used to produce the trajectories for movements such as those during the stance phase – this is not shown in the figure)**. Only a part of the controller is shown (only some of the existing behaviors and only for the right front leg). During normal behavior, the internal body model (upper left) serves perception. Switch 2 being in pos 1 provides proprioceptive input (e.g., joint angles from the legs). The body model may also receive external sensory input (e.g., from the antennae or visual input, arrow from above). If the system runs into a problem, the body model is, together with the procedural networks (Swing-net, Stance-net), used for trying out variations of behaviors. In this case both switches are flipped from position 1 to position 2 and the motor control (double-lined arrows entering switch 1 on the right) is routed, not to the body anymore, but to the body model (dashed double line). This circuit is used for internal simulation and predicts the sensory consequences of the action. The whole process is repeated until a suitable behavior has been found. For further explanations see text.

Instead, we will point to the fact that the property of this body model to act as a forward model can also be exploited for prediction. Whereas the term “prediction” usually describes the ability to provide expected sensory signals that can then be compared with actual sensor values (allowing, e.g., for correction of errors in the model), here we address another property. Internal simulation can also be used for prediction of “higher-level” expectations, for example, whether in a specific situation walking can be successfully continued. Together with the ability to exploit various elements of the motor memory, new kinds of procedures could be tested through internal simulation on being successful or not, thus allowing for the faculty of “internal trial-and-error.” In this sense, Schilling and Cruse (2008) have proposed a way of using the body model in cooperation with a procedural memory. Performing internal simulation is possible within this architecture when the output of the complete motor controller is not given to the body, i.e., the muscles (or in the case of a robot, the motors), but is instead directly projected back to the input of the body model. How this could be done is schematically illustrated in Figure 10. Only two procedural elements of one leg, the Swing-net and the Stance-net (for details see Dürr et al., 2004) of the right front leg, are depicted. The function of Swing-net has been explained earlier. The Stance-net is very simple as it contains only three Integral controllers, one for each joint. The reference values for these controllers are provided by the joint angles determined by the leg network (see The Leg Model). During normal walking, the output of these networks drives the leg muscles, as shown by switch 1 being in position 1. Proprioceptive feedback from the legs is given to the body model (switch 2 in position 1) which in turn provides information on joint angles to the procedures (Swing-net, Stance-net) thus closing the loop through the world. To allow the system to internally simulate a behavior, in our example simulate various ways of walking, both switches have to be moved to position 2. This causes the movement of the real body to stop. Instead the loop contains and drives only the body model and not the body. The more accurately the body model represents the properties of the real body (as well as selected properties of the environment, e.g., an obstacle), the better the simulated behavior corresponds to the behavior that would have been performed by the real body.

In our simulation approach, such an imagined behavior is elicited if during normal behavior a problem has been detected. A problem is characterized by a situation that cannot be handled by the currently performed behavior.

The network will, however, be able to find a solution to the problem only if the system can show some creativity. This means that new behaviors can be performed that are normally not elicited in the actual context. We assume that creativity is given by the faculty of the complete system to select new motivation units, i.e., procedural memory elements that are not activated in the actual context. We are currently working with a simple expansion of the motivation unit network to allow for creativity as characterized here (for more information see Schilling and Cruse, submitted).

If this approach turns out to be successful, we can distinguish between three levels of decision making (Cruse, 2009). The lowest level is characterized by a sensory-driven winner-take-all network, as for example is given in simple Braitenberg (1986) vehicles. The strongest sensory input determines which behavior will be performed (e.g., moving to stimulus A or to stimulus B). Noise plays merely a marginal effect as it will influence the decision only if both sensory inputs are very similar. A more complex “decision” structure would base its decision also on the current state of the system. In the case of our system the current state is represented by the motivation units as for example applied in the winner-take-all network controlling the swing-stance transitions. Both levels of decision making can be attributed to so-called reactive systems. The third level is characterized by the above mentioned system endowed with the property of internal simulation, i.e., with the ability to predict, in combination with the ability to test new behavioral solutions. As the search for new behavioral solutions, i.e., new procedures not used in the current context, is equipped with some stochasticity, an external observer cannot predict the new behavior invented by this system. This property, following the definition of Cleeremans (2005), may be characterized as comprising a volitional decision. In this way, the system is able to act in unknown and problematic situations. It is able to vary existing behaviors and, importantly, anticipates consequences of new behavioral plans before actually executing them. This allows the system to try different possible adaptations and select the one that predicts a desired outcome. The system uses the predictive capabilities of the internal model and becomes an anticipatory system following the definition of Pezzulo et al., (2008, p. 23) who provide a broad overview on anticipation and anticipatory artificial systems.

Möller and Schenck (2008) proposed another example of internal simulation to test if a specific behavior can be performed successfully. In their system, an inverse model is learned and required to suggest actions for a robot exploring a corridor with open and blocked doorways. Here, the robot acquired a forward model through active exploration. A sensorimotor representation has been constructed that is able to predict sensory consequences of a movement depending on the current state. Recruiting this forward model, the robot was able to internally simulate possible actions without actually performing them. An inverse model for selection of a suitable action was learned through ongoing internal simulations. Essentially, this model takes into account projected sensory consequences and only suggests actions that appear suitable in the long-run. Möller and Schenck (2008) relate this sensory representation to Gibson’s theory of affordances, which states that an object is not represented simply by what can be sensed, but in the way the object relates to the robot (Gibson, 1979). The approach of Möller and Schenck (2008) learns an inverse model for the selection of appropriate action commands. Importantly, the possible commands are quite simple and elegant, but, due to its simple body structure, do not need to involve sophisticated control of a complex robot consisting of multiple parallel and serial joints. For the case of a hexapod walker with many (22) DoF this will become much more complicated as the computation of inverse models itself has shown to be problematic in such cases. This is especially true because this computation is closely intertwined with the sensory representation and the prediction of the sensory values. We assume that only a larger structure like the MMC model proposed here, which tightly integrates inverse and forward models, allows exploiting the flexibility of the internal model to play around with variations of existing behaviors, and to come up with new behaviors that can be tested in internal simulation.

A number of articles address the question of planning ahead on an even more abstract level. For a typical and interesting example we will briefly refer to the work of Toussaint (2006). Starting from Hesslow’s (2002) notion of internal simulation as an activation of motor structures while suppressing execution, the core idea is that perceptions can be predicted as a simulation that directly leads to perceptual consequences. Central to their system is a sensorimotor map that couples sensor and motor signals in a joint representational layer. This layer is modeled as laterally connected neural layers (there are specific layers for the sensory representation and the motor commands as well as an intermediate layer coupling the two). In the same way as in the MMC network, a current state is represented through the activation of the network. The network can be driven by activations. In this way, anticipation is realized as the shifting of activity in the network triggered by external modulations provided by the motor commands. Toussaint (2006) used this network to demonstrate planning capabilities. The task was to navigate a maze. Initially, a sensorimotor map is learned through random explorations that represent the maze environment. Afterward, in the planning phase a goal stimulus is applied to the network that represents the goal position. This activation spreads through the network constrained by the topography of the maze as represented in the different networks. Here, the back and forth between sensory and motor network basically correspond to predicting sensory consequences of motor actions. For possible movements (the way is not blocked) the motor activation is maintained and can further spread. When in contrast a movement is predicted as not possible the motor activation is inhibited and here the spreading stops. In this way the networks explore the different possibilities. Although the work of Toussaint (2006) deals with even simpler motor commands than the approach of Möller and Schenck (2008), their work shows nicely how the idea of internal simulation can be understood in terms of neural computation and can be based on the spreading of activation. At the same time it demonstrates how this relies on the close coupling of sensory and motor representation and especially that this approach requires transformation mediating in both directions.

## Conclusion and Future Work

Anticipation of effects of action is crucial to motor control, but it is also a prerequisite for planning (Clark, in press). We have described an approach using an artificial RNN that constitutes an internal model of the body. The model is flexible and can address diverse tasks: We have shown how it can be used in motor control for targeted movements. But the model is also predictive in its nature. It is able to anticipate the effects of action and we have demonstrated how the model can estimate the resulting posture when a movement is executed. While we focused on joint position information, an extension of the model can be used to integrate dynamic influences and control signals like velocities. Following such an approach leads to natural and biological movement characteristics (Schilling, 2011a).

The model is a holistic model and as such it can be flexibly applied in other contexts serving other functions as well. We have used the model in perception in past work and used it during the observation of movements to reconstruct the observed movement (Schilling, 2011b).

Finally, we have explained how the internal model can be introduced in a robotic control structure for a hexapod robot and have briefly illustrated how the predictive capabilities can be exploited by the system in order to anticipate the effects of action before actually carrying out an action. This allows the controller to evaluate the consequences of an action and decide against performing it when it turns out to be dangerous.

Currently, we are realizing this control structure for a hexapod walker. As of now, the body model is applied in the stance controller. It is used in a similar way as described in Section “Application as an Inverse Model” (see also Schilling et al., in press). As the body model is already part of the control loop, we are going to extend the model and introduce additional redundant sensory information that is available on the real robot. As the model realizes an integration principle it can be used to fuse the sensory information of different modalities.

The control structure will be extended as explained above to account for new problematic situations to which none of the present motor primitive can react. Due to the predictive capabilities of the body model, the body model, it can be used for internal simulation. The controller can differentiate between different alternatives and variations of behaviors using their outcomes. In this way, the system can plan its action and becomes cognitive. The system takes the outcome of action into account to decide about future action. Even though the internal model is not what has changed in the system when becoming cognitive, the internal model of the body is the central part of the cognitive system. The predictive capabilities are crucial and it is the flexibility of the proposed internal body model that allows the model to be recruited in planning ahead (Anderson, 2010).

In the future, the control structure shall learn these new successful behaviors and integrate them into the overall controller structure which means that the new behaviors will also take part in the process of action selection. The model of the body is a central representation, but it is only a starting point. Even the simple body model relates to some parts of the environment where the tarsi are touching the ground. For example, the spatial arrangement of the foot points of the body model provide a simplistic representation of the environment in a way that is relevant to the animal and it’s action. Our bottom-up approach allows introduction of such higher-level representations as grounded internal models as they are not detached from the lower levels of motor control. Instead, the higher levels of representation can be tightly interconnected and directly anchored in the lower levels of body representation.

## Conflict of Interest Statement

The authors declare that the research was conducted in the absence of any commercial or financial relationships that could be construed as a potential conflict of interest.

## Figures and Tables

**Figure A1:**
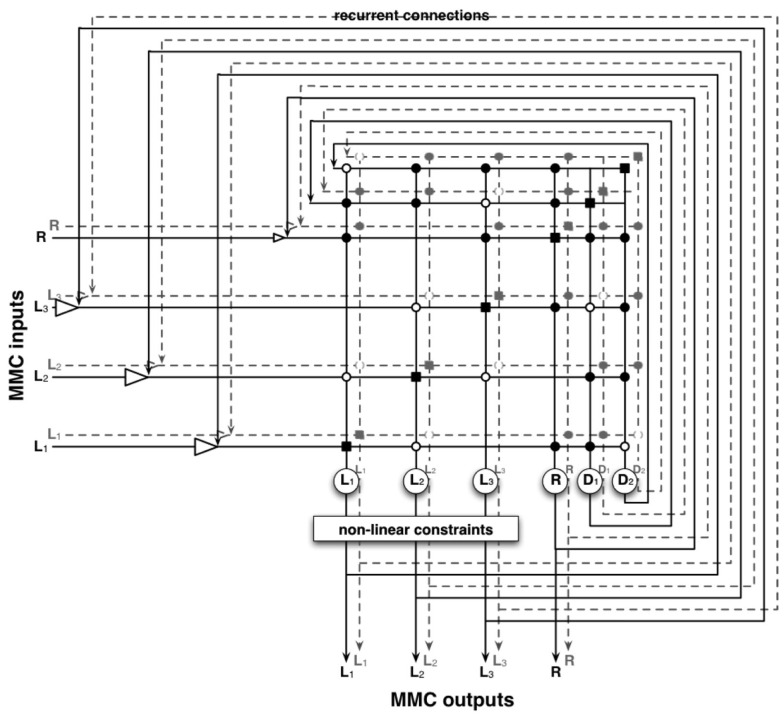
**The recurrent neural network consisting of 2 × 6 units**. The network is composed of two identical networks (The one for the *x*-components is shown with black lines and the other for the *y*-components of the vectors is shown with the dashed lines). The units represent the components of the six vectors *L*_1_, *L*_2_, *L*_3_, *D*_1_, *D*_2_, and *R* of the manipulator. When an input is given, the corresponding recurrent channel is suppressed (symbolized by the open arrow heads).

## Appendix

### Classical MMC network describing a three-segmented arm working in a two-dimensional plane

Figure 1 shows the manipulator that consists of three segments, upper arm *L*_1_, lower arm *L*_2_, and hand *L*_3_, controlled by three joints: shoulder, elbow, and wrist joint. The shoulder is situated at the origin of the *x*–*y* coordinate system. In addition, we introduce diagonal vectors *D*_1_ and *D*_2_ and an end-effector vector *R* that points to the tip of the arm. In the following we explain how this vector graph (as shown in Figure 1) can be used to derive equations. The resulting set of equations constitutes a weight matrix for a RNN. First, we determine all equations formed by all possible combinations of vectors forming vector triangles: The complete graph consists of several such triangles. Each triangle is a closed polygon chain which means that the three vectors complement each other to zero.

$$\begin{array}{rcll} L_{1}+D_{2}-R=0 & & & \text{(A1)}\\ L_{1}+L_{2}-D_{1}=0 & & & \\ D_{1}+L_{3}-R=0 & & & \\ L_{2}+L_{3}-D_{2}=0 & & & \end{array}$$

Any of these equations can be solved for each of the contained variables. Next, all equations determining a given variable are used to form a set of equations. In this simple example, each variable can be found in two equations. In (2) this is shown for *L*_1_ as an example.

$$\begin{array}{rcll} L_{1}=R-D_{2} & & & \text{(A2)}\\ L_{1}=D_{1}-L_{2} & & & \end{array}$$

In this way, we obtain six systems of two equations each. This procedure is called Multiple Computation of the same variable.

As we are considering a dynamic system that is expressed with respect to time, all variables depend on the time. The MMC principle is an iterative procedure to calculate new values for the next time step depending on the current state. For each variable the two equations are simply integrated through calculation of the Mean value (therefore the name – MMC).

(A3) $$L_{1}(t+1)=\frac{1}{2}(R(t)-D_{2}(t))+\frac{1}{2}((D_{1}(t)-L_{2}(t))$$

The result is one equation describing the new value for a variable depending on a weighted sum of the current values of the other variables. These equations can be directly understood as a weight matrix for a neural network. To establish the weight matrix, the vectors have to be decomposed into their *x*- and *y*-components. This leads to a set of corresponding linear equations. In the 2D example, we get two identical nets (one for each component) and for an extension to three dimensions we only have to introduce a third network representing the *z*-component. The network is shown in Figure A1.

The introduction of recurrent connections, i.e., feeding back the current value of the variable weighted by a damping factor, leads to smoother transitions in the network. The network becomes more stable and oscillations are prevented.

(A4) $$\begin{array}{c} L_{1}(t+1)=\frac{d}{d+2}L_{1}(t)+\frac{1}{d+2}(R(t)-D_{2}(t))\\ +\frac{1}{d+2}((D_{1}(t)-L_{2}(t)) \end{array}$$

Until now, we described the simple linear version of the classical MMC approach. All variables are allowed to freely change. For the rigid segments of the arm (the upper and lower arm as well the hand) we usually want to constrain the changes of variables, e.g., the segments shall not change length or the joint movements shall be restricted. This can be easily done through introducing constraints and applying the constraints after each calculation (Steinkühler and Cruse, 1998). To evade the introduction of non-linear constraints one can also use other kinds of representation. When using joint angle representation, it is not necessary to normalize the segments length after each time step. We have shown such a solution for general movements of a nine DoF arm in three dimensions using dual quaternion representations (Schilling, 2011a). In the following, we want to derive a simpler network for the special case of the insect leg.

### Angular MMC network representing an insect leg

The leg of a stick insect only consists of three DoF (see Figure 3). Therefore, it is possible to derive a simple MMC network using redundant trigonometric relationships. The leg model can compute inverse and forward kinematics of the manipulator.

Due to the kinematic structure we can derive a specific solution for the insect leg. As the second and third joint act in a plane and their rotation axes are parallel, we can use basic trigonometric function to come up with a solution for these joint angles (see in Figure 3C) which hold true in this plane. We can compute the forward kinematics for the leg. The height value directly corresponds to the z value given in the leg coordinate system:

(A5) $$z=s_{1}\sin\beta +s_{2}\sin\left(\beta +\gamma \right)$$

The width (as a numeric value) is given as the projection of the leg onto the leg plane.:

(A6) $$l=s_{o}+s_{1}\cos\beta +s_{2}\cos\left(\beta +\gamma \right)$$

Both values are computed as a summation of the single segment portions. From this we can derive multiple computations related to the joint angles of the second and third joint. We end up with sine and cosine expression for the angles.

$$\begin{array}{rcll} & \sin\beta =\frac{z-s_{2}\sin\left(\beta +\gamma \right)}{s_{1}} & & \text{(A7)}\\ & \cos\beta =\frac{l-s_{0}-s_{2}\cos\left(\beta +\gamma \right)}{s_{1}} & & \end{array}$$

As sine and cosine functions are only surjective, the inverse is ambiguous and cannot directly be used to calculate the actual angles. But we can combine these and at the same time integrate the two equations by using the arc tangent function which is the quotient of the two. Again, the variables are time dependent and as we use the arc tangent to integrate the multiple computations the result is the new value for a variable:

(A8) $$\beta \left(t+1\right)=\arctan\frac{z\left(t\right)-s_{2}\sin\left(\beta \left(t\right)+\gamma \left(t\right)\right)}{l\left(t\right)-s_{0}-s_{2}\cos\left(\beta \left(t\right)+\gamma \left(t\right)\right)}$$

We can derive an equation for representing gamma in the same way.

The first joint is perpendicular to the other two joints. The axis of rotation lies in the leg plane and coincides with the *z*-axis of the leg coordinate system. Therefore, the rotation can be directly computed from the *x-* and *y*-values of the leg vector (see Figure 3B), showing a view directly from above).

(A9) $$\tan\alpha =\frac{y}{x}$$

We can also setup an additional equation for the projection of the leg onto the leg plane.

(A10) $$\sin\alpha =\frac{y}{l}$$

The multiple computations can now be used to calculate the different variables which are then integrated. On the one hand, as described above, we integrate several of the trigonometric relations into one equation through application of the arc tangent function. On the other hand, we integrate multiple computations for one variable as the computation of the mean value of the – possibly – different solutions. Here, we also include the preceding value of the variable weighted by a damping factor in order to avoid oscillations. Again, the resulting set of equations can be directly interpreted as a RNN weight matrix.

This network has several advantages compared to an explicit computation. First, the network is able to solve forward, inverse, and any mixed kinematic problems in a few iteration steps. Second, as explicit computations involve the application of the inverse of sine and cosine functions, these require case distinctions. Third, for cases where no solution is possible (e.g., the target point is too far away), the net still converges to a stable and geometric valid solution which is minimizing the error (in the example this would be the leg pointing into the direction of a far away target).
